# Therapeutic Uses of Rituximab and Clinical Features in Immunoglobulin G4-Related Disease: A Systematic Review

**DOI:** 10.7759/cureus.45044

**Published:** 2023-09-11

**Authors:** Utkarsh Patel, Ayushi Saxena, Dhara Patel, Ismat E Ayesha, Neetha R Monson, Nimra Klair, Ann Kashmer Yu

**Affiliations:** 1 Internal Medicine, California Institute of Behavioral Neurosciences & Psychology, Fairfield, USA

**Keywords:** autoimmune, igg4 autoimmune disease, glucocorticoids, corticosteroids, rituximab, igg4-related disease

## Abstract

This research presents a systematic review focusing on rituximab's therapeutic applications in immunoglobulin G4 (IgG4)-related disease (IgG4-RD), a rare condition characterized by immune-mediated systemic inflammation and tissue fibrosis, as well as the clinical features of IgG4-RD. While the disease commonly affects organs such as the bile ducts, lymph nodes, retroperitoneum, pancreas, and salivary glands, it can potentially involve other organs. This intricacy often leads to diagnostic challenges due to clinical overlaps with cancer, infections, and other autoimmune disorders.

The diagnosis of IgG4-RD necessitates a comprehensive approach involving laboratory tests, imaging studies, and clinical assessments. Symptoms can vary, ranging from lymphadenopathy to jaundice, affecting multiple organs. Although elevated blood IgG4 levels and findings of tissue involvement and fibrosis on imaging can be suggestive, they lack the specificity for a definitive diagnosis. Early diagnosis is crucial for initiating corticosteroids and immunosuppressive to prevent further damage from IgG4-RD. This study highlights the therapeutic role of rituximab in managing this condition.

Adhering to the Preferred Reporting Items for Systematic Reviews and Meta-Analyses (PRISMA) criteria, the research identifies and evaluates relevant literature across various electronic databases, including PubMed, ScienceDirect, and Google Scholar. This review includes 14 selected publications, comprising three systematic reviews, three observational studies, four narrative reviews, and four case reports. The study design ensures a comprehensive evaluation of rituximab's potential efficacy in treating IgG4-RD and its associated clinical characteristics. Based on this study, it can be concluded that IgG4-RD can potentially be treated with rituximab, particularly in cases of relapse and maintaining remission.

## Introduction and background

Immunoglobulin G4 (IgG4)-related disease (IgG4-RD) is a rare disorder characterized by IgG4-positive plasma cell infiltration and tissue fibrosis due to immune-mediated systemic inflammation [1]. It is an immune-mediated disorder characterized by dysregulated immune responses leading to organ-specific pathology. It predominantly affects middle-aged to elderly individuals, with a male preponderance, although rare cases occur in children. IgG4-RD often involves multiple organs simultaneously, impacting two to six organs.

The lymph nodes, bile ducts, retroperitoneum, pancreas, and salivary glands are typical sites of involvement; however, it may affect almost any organ system [1]. Clinical similarities between IgG4-RD and cancer, infection, and other autoimmune illnesses make diagnosis difficult. Multiple evidence, such as laboratory tests, imaging scans, and clinical observations, must come together to confirm a diagnosis of IgG4-RD [2]. Symptoms might include jaundice and stomach pain in cases involving the bile ducts or swelling and pain in the salivary glands [3]. Due to the possibility of incorrect or delayed diagnosis brought on by heterogeneity, a comprehensive strategy is required. Blood IgG4 levels that are above average are suggestive but not definitive. Tissue involvement and fibrosis may be shown in CT and MRI studies [4]. These findings do not provide sufficient diagnostic specificity for IgG4-RD. The proper diagnosis of IgG4-RD requires a team effort to examine clinical symptoms, test results, imaging reports, and histopathology. This coordinated approach is crucial for the early administration of corticosteroids and immunosuppressive medicines, which mitigate inflammation and tissue damage, respectively.

IgG4-related disease is still relatively under-recognized and often misdiagnosed due to its diverse and overlapping clinical manifestations, which can mimic a variety of other conditions [5]. This diagnostic challenge is exacerbated by the fact that there is no single definitive test or gold standard for diagnosing IgG4-RD. Instead, a combination of clinical, laboratory, radiological, and histopathological criteria must be meticulously evaluated to arrive at a confident diagnosis [6]. Given the potential for misdiagnosis, the importance of a comprehensive and multidisciplinary diagnostic approach cannot be overstated.

In addition to the typical organ involvement mentioned, such as the bile ducts, retroperitoneum, pancreas, and salivary glands, IgG4-RD has been found to affect an expanding array of organ systems, including the thyroid, lung, kidney, and even the central nervous system [7]. This wide-ranging spectrum of potential manifestations further underscores the need for a holistic diagnostic strategy. Moreover, the disease can present with subtle and nonspecific symptoms, making it challenging for healthcare providers to distinguish it from other conditions. Patients might present with fatigue, weight loss, or non-specific pain, which can easily be mistaken for other systemic disorders [2]. Consequently, a heightened awareness of IgG4-RD among medical professionals is needed to ensure accurate and timely diagnosis, thereby preventing potential complications and irreversible organ damage.

Rituximab is a monoclonal antibody that targets a specific protein called CD20 found on the surface of B cells. It is primarily used in the treatment of several autoimmune disorders and certain types of malignancies, particularly B-cell lymphomas and leukemia. Rituximab works by binding to CD20-positive cells and leading to the destruction of B cells, which play a crucial role in immune responses and the production of antibodies. Rituximab helps suppress unwanted immune responses by reducing the number of memory B cells in circulation.

Rituximab has emerged as a promising therapeutic option for IgG4-RD in recent years, showing the potential in inducing and maintaining remission. Rituximab, a monoclonal antibody that targets CD20-positive B cells, has demonstrated efficacy in various autoimmune conditions [8]. Its mechanism of action in IgG4-RD involves the depletion of B cells, which play a key role in the pathogenesis of the disease by producing excess IgG4 antibodies and contributing to inflammation. As IgG4-RD is a complex and multifaceted disorder, the use of rituximab represents a significant advancement in treatment, offering an alternative for patients who do not respond well to conventional treatments like corticosteroids or immunosuppressive agents. This systematic review aims to comprehensively assess the clinical features of IgG4-RD, highlight the challenges in its diagnosis, and thoroughly evaluate the therapeutic efficacy and potential of rituximab as a valuable addition to the treatment armamentarium for this intricate and enigmatic disorder.

## Review

Methods

Study Objective

This study seeks to provide a detailed analysis of the effects of rituximab in IgG4-RD and its clinical characteristics. Our purpose of the study is to evaluate rituximab’s use in treating IgG4-RD while also pinpointing any blind spots in our knowledge. This comprehensive literature evaluation also examines the commonly associated clinical features of IgG4-RD.

Study Design

The studies selected for evaluation were thoroughly filtered using inclusion and exclusion criteria. Additionally, the studies were chosen based on their relevance to the research's objectives, including keywords such as "IgG4-related disease," "rituximab," and "glucocorticoids." The Preferred Reporting Items for Systematic Reviews and Meta-Analyses (PRISMA) criteria were used to conduct this systematic review [8-10].

Inclusion and Exclusion Criteria

The inclusion criteria of this research include studies that analyze the effect of rituximab on IgG4-RD and its clinical characteristics. This encompasses diverse study designs (observational studies, case reports, systematic and narrative reviews) reporting on clinical outcomes, adverse events, response rates, and other relevant factors. These studies must be published in English within the last 10 years (2013-2023) and exclusively involve human participants.

The criteria for exclusion involve the removal of studies involving animals, in vitro experiments, or phase 1 clinical trials. Additionally, studies that do not focus on rituximab or IgG4-RD, those lacking adequate data regarding clinical features or treatment effects, studies not in English, and those scoring below 70% in quality assessment are also to be excluded.

Data Extraction

Three databases, including PubMed, Science Direct, and Google Scholar, were used to retrieve the data from the studies necessary for this research. The information that was collected consists of research specifics (author, publication year, study design), patient specifics (sample size, age, treatment duration), clinical outcomes (response rates, relapse, and remission rates), clinical features, and adverse events.

Search Strategy

PubMed, Science Direct, and Google Scholar were the three electronic databases combed for relevant articles. Keywords “rituximab,” “glucocorticoids,” and “IgG4-related disease” will all be part of the search approach. Only articles written in English that were published before May 6, 2023, will be included in the search. When research studies from the chosen databases were combined and duplicates were removed, the search technique yielded a total of 323 results. The field searches utilized in the process were meticulously chosen based on the keywords used in the previous literature and through Medical Subject Headings (MeSH), depending on the database used.

The records were initially examined based on the titles and abstract, and irrelevant studies were excluded. Microsoft Excel (Microsoft Corporation, Redmond, WA) was used for duplicate removal and organizing each reference in the order. EndNote citation manager (Clarivate, London, UK) was used to convert and organize references to the desired format. The outcomes of the search strategies are unambiguously illustrated in Table 1.

**Table 1:** Search strategy and results MeSH: Medical Subject Headings.

Databases	Date of search	Keywords	Filters	Search strategy	Search results
PubMed	May 3, 2023	Immunoglobulin G4-related disease, Rituximab, Glucocorticoids	Last 10 years, full text, English language, humans	#1 Immunoglobulin G4-Related Disease OR IgG4 Related Systemic Disease OR IgG4-Related Disease OR IgG4 Related Kidney Disease OR Immunoglobulin G4-Related Kidney Disease OR IgG4 Associated Autoimmune Disease OR IgG4-Associated Autoimmune Diseases OR ("Immunoglobulin G4-Related Disease"[Majr]) #2 Rituximab OR CD20 Antibody OR RITUXAN OR monoclonal antibodies OR monoclonal antibody OR ("Rituximab"[Mesh]) #3 Glucocorticoids OR steroid OR steroids OR Prednisone OR corticosteroid OR corticoid OR Cortisone OR prednisolone OR betamethasone OR dexamethasone OR hydrocortisone OR triamcinolone OR Methylprednisolone OR Dexamethasone OR Cortef OR Medrol OR Depo-Medrol OR ("Glucocorticoids"[Majr]) #1 AND #2 AND #3	215
Google Scholar	May 3, 2023	Rituximab, IgG4-related disease, Glucocorticoids	2013-2023	"Rituximab" AND "IgG4 related disease" AND "Glucocorticoids	1760
ScienceDirect	May 6, 2023	Rituximab, IgG4-related disease, Glucocorticoids	2013-2023, research articles, medicine and dentistry	"Rituximab" AND "IgG4 related disease" AND "Glucocorticoids"	148

Risk of Bias in Individual Studies

The quality and risk of bias of included studies are assessed using appropriate tools such as the Newcastle-Ottawa Scale for observational studies [11], Scale for the Assessment of Narrative Review Articles 2 (SANRA) for narrative review articles [12], Assessment of Multiple Systematic Reviews 2 (AMSTAR 2) for systematic reviews [13], and Joanna Briggs Institute (JBI) Critical Appraisal Checklist for case reports [14]. Two independent reviewers assessed the quality of each study for accuracy, and any disagreements were resolved through discussion and consultation with a third reviewer.

Results

**Figure 1:**
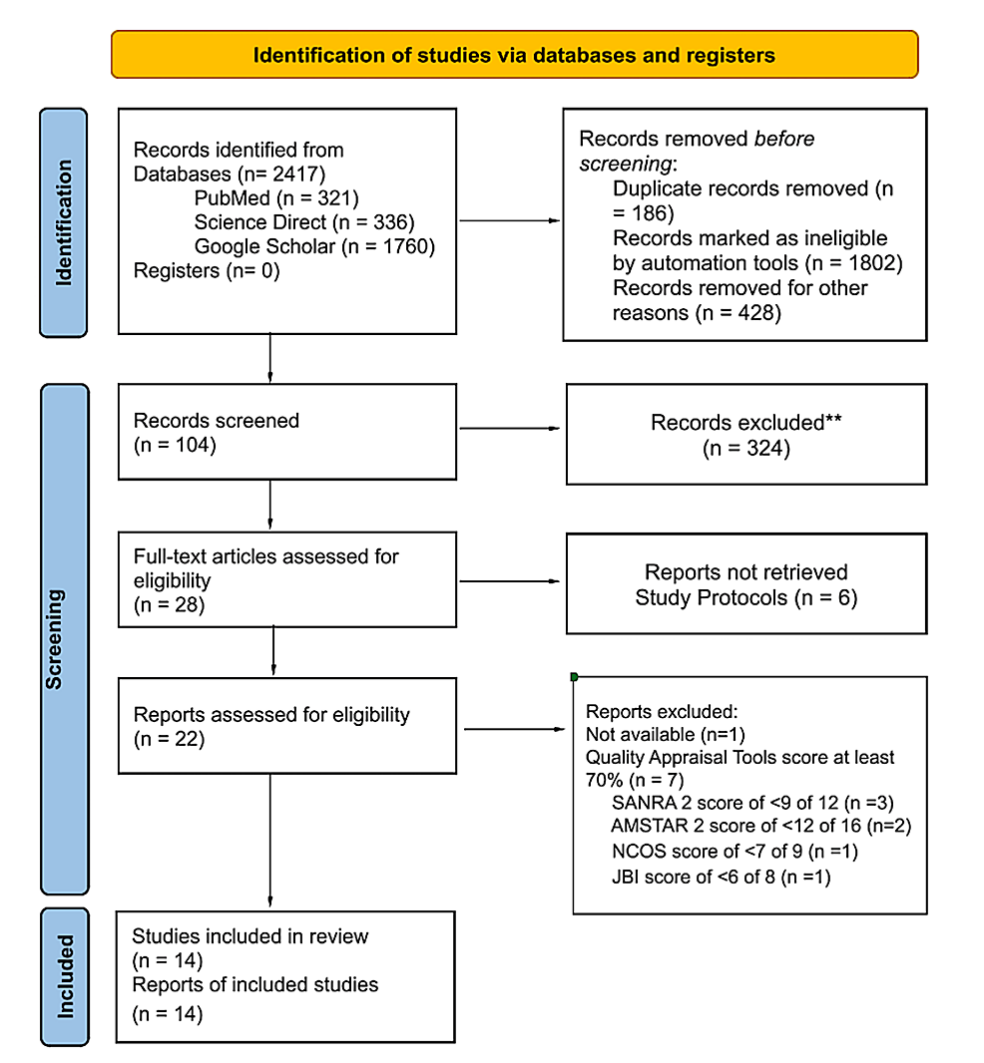
PRISMA flowchart of the study selection PRISMA: Preferred Reporting Items for Systematic Reviews and Meta-Analyses; JBI: The Joanna Briggs Institute critical appraisal tools; NCOS: Newcastle-Ottawa Scale; AMSTAR 2: Assessment of Multiple Systematic Reviews 2; SANRA 2: Scale for the Assessment of Narrative Review Articles 2.

We conducted a systematic review to explore rituximab's therapeutic use in IgG4-RD and its clinical features. After a comprehensive search of relevant literature, we identified 22 studies that met our inclusion criteria. These studies were published between 2013 and 2023 and encompassed various aspects of IgG4-RD and rituximab treatment. We started with 104 potentially relevant research, assessed the quality of 22 publications, and ultimately included 14 in our systematic review since they matched our inclusion criteria. A total of 14 publications were used to produce the study's findings: three systematic reviews, three observational studies, four narrative reviews, and four case reports. No other resources were added. The summary of the study selection and study designs is presented in an organized manner in Figure 1.

Quality Assessment

Two independent reviewers screened the titles and abstracts of retrieved articles and did a quality assessment check to identify potentially relevant studies. We conducted a comprehensive evaluation of all studies that could meet the criteria for inclusion. To ensure accuracy and fairness, any discrepancies that arose were addressed either by reaching a consensus or consulting with an impartial third reviewer. This approach allowed for a comprehensive evaluation of the systematic review, resulting in relevant data. The quality appraisal score for each study is shown in Table 2, using the appropriate evaluation technique for each type of study.

**Table 2:** Results of quality appraisal * It is important to note that studies scoring below 70% in the quality assessment were excluded from the study selection. Scores reflect the assessment of the studies' quality by the respective authors and evaluation tools used. JBI: The Joanna Briggs Institute critical appraisal tools; NCOS: Newcastle-Ottawa Scale; AMSTAR 2: Assessment of Multiple Systematic Reviews 2; SANRA 2: Scale for the Assessment of Narrative Review Articles 2; N/A: not available.

Study title	Study type	Tool	Author 1 (%)	Author 2 (%)	Average (%)
Diagnostic and treatment workup for IgG4-related disease [15]	Narrative review	SANRA	83.33%	83.33%	83.33%
IgG-4 related disease: An introduction [16]	Narrative review	SANRA	50.00%	58.33%	54.17%
Immunoglobulin G4-related disease: An update [17]	Narrative review	SANRA	83.33%	41.67%	62.50%
Update on classification, diagnosis, and management of immunoglobulin G4-related disease [18]	Narrative review	SANRA	83.33%	83.33%	83.33%
IGG4-related disease: clinical and laboratory features in one hundred twenty-five patients [19]	Narrative review	SANRA	83.33%	100.00%	91.67%
Recent advances in understanding and managing IgG4-related disease [20]	Narrative review	SANRA	66.67%	66.67%	66.67%
B cell targeted therapy for immunoglobulin G4-related disease [21]	Narrative review	SANRA	83.33%	66.67%	75.00%
IgG4-related disease: Advances in the diagnosis and treatment [22]	Systematic review	AMSTAR	50.00%	37.50%	43.75%
Therapeutic approach to IgG4-related disease: A systematic review [23]	Systematic review	AMSTAR	75.00%	81.25%	78.13%
Systematic review of safety and efficacy of rituximab in treating immune-mediated disorders [24]	Systematic review	AMSTAR	76.92%	100.00%	88.46%
IgG4-related disease: Is rituximab the best therapeutic strategy for cases refractory to conventional therapy? Results of a systematic review [25]	Systematic review	AMSTAR	25.00%	72.73%	48.87%
Glucocorticoids and steroid sparing medications monotherapies or in combination for IgG4-RD: a systematic review and network meta-analysis [26]	Systematic review	AMSTAR	71.43%	93.75%	82.59%
Serum IgG4 level during initial treatment as a predictor of relapse in IgG4-related disease [27]	Observational	NCOS	75.00%	100.00%	87.50%
Predictors of disease relapse in IgG4-related disease following rituximab [28]	Observational	NCOS	87.50%	75.00%	81.25%
Failure of remission induction by glucocorticoids alone or in combination with immunosuppressive agents [29]	Observational	NCOS	75.00%	87.50%	81.25%
Improved clinical outcomes of tocilizumab versus cyclophosphamide for IgG4-related disease [30]	Observational	NCOS	62.50%	75.0%	68.75%
Successful treatment of tubulointerstitial nephritis in immunoglobulin G4-related disease with rituximab [31]	Case report	JBI	75.00%	87.50%	81.25%
Successful treatment of IgG-4 related sclerosing disease with rituximab: a novel case report [32]	Case report	JBI	75.00%	100.00%	87.50%
IgG4-related disease with cutaneous manifestations treated with rituximab: case report and literature review [33]	Case report	JBI	100.00%	87.50%	93.75%
Rituximab for corticosteroid-resistant relapsing IgG4-related intracranial pachymeningitis: report of two cases [34]	Case report	JBI	62.50%	100.00%	81.25%
Two cases of refractory IgG4-related disease successfully treated with tocilizumab [35]	Case report	JBI	N/A	N/A	N/A
Dupilumab as a novel steroid-sparing treatment for IgG4-related disease [36]	Case report	JBI	87.50%	100.00%	93.75%

The table includes studies that were categorized by the type of study and the evaluation tool to assess their quality. Each study has the average score in percentage given by author 1 and author 2 and the combined average score of both authors.

Narrative reviews: These studies provide an overview or update on IgG4-RD. The average scores range from 54.17% to 83.33%, indicating moderate to high quality.

Systematic reviews: These studies systematically evaluate the existing literature on IgG4-RDs. The average scores range from 43.75% to 82.59%, indicating moderate quality.

Observational studies: These studies observe and analyze IgG4-RDs in real-world settings. The average scores range from 81.25% to 87.50%, indicating high quality.

Case reports: These studies present individual cases of IgG4-RD and their treatment outcomes. The average scores range from 81.25% to 93.75%, indicating high quality.

Clinical features

The IgG4-RD is recognized as a multi-organ fibroinflammatory disorder with a wide range of affected organs. The involvement of glands, such as salivary and submandibular glands, lymphadenopathy, and epithelial edema, which are essential clinical markers for diagnosis. Additionally, autoimmune pancreatitis is common in IgG4-RD characterized by pancreatic enlargement, lymphoplasmacytic infiltrates, and fibrosis. This condition often presents with jaundice and biliary strictures, indicating the potential involvement of the biliary tract. The clinical features of IgG4-RD described in Table 3 highlight this condition's diverse and systemic nature.

**Table 3:** Clinical features of IgG4-related disease

Organ involvement	Clinical features	
Glands and lymphatic system		Lymphadenopathy, salivary gland edema, submandibular gland edema
Autoimmune		Autoimmune pancreatitis, lymphoplasmacytic infiltrates, retroperitoneal fibrosis, sclerosing cholangitis, elevated serum IgG4 levels
Biliary tract		Obstructive jaundice, cholangitis, and biliary strictures
Ocular		Proptosis, ophthalmoplegia, ocular edema, and dacryoadenitis
Skin		Skin nodules, plaques, subcutaneous masses, and eczema
Renal		Tubulointerstitial nephritis, renal mass, and renal dysfunction
Lungs		Asthma, bronchitis, interstitial lung disease, pulmonary nodules and pleural effusion

Additionally, IgG4-RD can also affect the orbit resulting in ophthalmoplegia, proptosis, ocular edema, and vision impairment. Dermatological involvement presents complexity to the disease presentation with manifestations such as erythema, nodules, plaques, masses, and eczema. The kidneys are also critically affected by this disease, often leading to tubulointerstitial nephritis, renal masses, and reduced kidney function. This autoimmune disease can also involve the respiratory system and may contribute to asthma, bronchitis, pulmonary nodules, pleural effusion, and interstitial lung disease.

In summary, the studies we examined in our review offer valuable insights into how rituximab affects IgG4-RD and its clinical characteristics. Lymph nodes, pancreas, and salivary glands are found to be the most commonly involved organs. However, the current evidence indicates that rituximab is a promising treatment option, but additional high-quality research, including randomized controlled trials, is required to determine its effectiveness, optimal dosage, and long-term results.

Therapeutic use of rituximab in IgG4-related disease

The studies in our review covered a wide range of topics related to IgG4-RD and rituximab, including disease introduction, safety, efficacy, diagnostic, and treatment workup. According to the available evidence, rituximab could be a treatment of choice for IgG4-RD. However, it is important to acknowledge that the studies included in this analysis differed in their design, patients they involved, treatment plans used, and duration for follow-up. Due to this variability in studies, it becomes challenging to draw definitive conclusions.

Among the included studies, several explicitly focused on rituximab treatment in IgG4-RDs. They examined the efficacy and safety of rituximab as a monotherapy or in combination with other medications. One study by Wang et al. investigated the combination therapy of leflunomide and glucocorticoids for remission in IgG4-RDs [37]. Another study by Wallace et al. explored predictors of disease relapse in IgG4-RD following rituximab treatment [28].

The findings from the included studies demonstrate that rituximab showed promising results in treating IgG4-RDs. It was associated with improved clinical outcomes, including remission induction, relapse prevention, and reduced disease activity. Furthermore, rituximab was generally well-tolerated, with only a few reported adverse events. The findings from these studies are included in Table 4.

**Table 4:** Conclusions from the selected studies

Study	Use of rituximab	Effects of rituximab
Jalilian et al., 2014 [33]	Therapeutic option	Improvement in cutaneous manifestations and reduction in IgG4 levels.
Gillispie et al., 2015 [32]	Therapeutic option	Improvement in IgG4-related sclerosing disease.
Wallace et al., 2015 [19]	Therapeutic option	Improvement in symptoms, reduction in IgG4 levels, and decreased disease activity.
Wallace et al., 2016 [28]	Therapeutic option	Reduction in disease relapse and sustained remission in some patients.
Brito-Zerón et al., 2016 [23]	Therapeutic option	Variable response, with some cases showing improvement in symptoms and reduction in IgG4 levels.
Abraham and Khosroshahi, 2017 [15]	Therapeutic option	Improvement in symptoms, reduction in IgG4 levels, and decreased disease activity.
Mageau et al., 2018 [34]	Therapeutic option	Improvement in corticosteroid-resistant relapsing intracranial pachymeningitis, improvement in symptoms, and reduction in disease activity.
Wang et al., 2018 [29]	Therapeutic option	Limited efficacy as a monotherapy, but may be practical with other immunosuppressive agents. Limited success in remission induction.
Kaegi et al., 2019 [24]	Therapeutic option	Effective in treating immune-mediated disorders, including IgG4-related disease, with a good safety profile.
Eroglu et al., 2019 [31]	Therapeutic option	Resolution of tubulointerstitial nephritis and improvement in renal function.
­­­­­­­Omar et al., 2020 [26]	Therapeutic option	Effective in reducing disease activity and steroid-sparing effect.
Yamamoto, 2021 [21]	Therapeutic option	Effective in suppressing disease activity and inducing remission in IgG4-related disease improvement in organs involved.
Chen et al., 2022 [18]	Therapeutic option	Improvement in clinical symptoms and reduction in serum IgG4 levels.
Choi et al., 2023 [27]	Therapeutic option	Reduction in disease activity and prevention of relapse.

Based on the selected studies, the advantages of using rituximab as a treatment for IgG4-RD are presented below.

Efficacy and Tolerability of Rituximab

Rituximab has shown promise in achieving remission and reducing relapse rates in IgG4-RD [26]. It has been associated with successful outcomes in various organ systems affected by IgG4-RD, including the kidneys, central nervous system, and skin [30-34]. Rituximab has been generally well-tolerated with a favorable safety profile in patients with IgG4-RD [24]. Serum IgG4 levels during initial rituximab treatment have been suggested as a predictor of relapse in IgG4-RD [27].

Rituximab vs. Glucocorticoids

Rituximab and glucocorticoids are both commonly utilized treatments for several autoimmune and inflammatory conditions. Glucocorticoids are the first line of treatment in most autoimmune disorders, including IgG4-RD. Although, rituximab is often considered when glucocorticoids alone fail to induce remission [29]. Combination therapy with rituximab and glucocorticoids has shown positive results in achieving remission and reducing relapse rates [26].

Rituximab vs. Other Immunosuppressive Agents

Rituximab was the most often utilized therapy after glucocorticoids when compared with other treatment options for IgG4-RD. Rituximab is considered a treatment option when immunosuppressive agents are ineffective or not well-tolerated [24]. It has shown efficacy in cases where other immunosuppressive agents have failed to induce remission [29].

In summary, rituximab appears to be a promising therapeutic option for IgG4-RDs, offering both efficacy and tolerability. It has shown potential benefits over glucocorticoids and other immunosuppressive agents, particularly in cases where these treatments have not provided adequate results.

Discussion

Rituximab's mechanism of action, targeting CD20-positive B cells and modulating the immune response, aligns with the underlying immunopathology of IgG4-RD [21,24]. It has also shown potential as a steroid-sparing agent and a treatment option for corticosteroid-resistant cases [4,25]. Several immunosuppressive agents, such as azathioprine, mycophenolate mofetil, methotrexate, and rituximab, have been used as steroid-sparing agents to achieve and maintain remission in IgG4-RD. While glucocorticoids still remain the first line treat for this autoimmune disease.

This medication is administered intravenously and it is typically given in multiple doses spread over several weeks or months. Common side effects include infusion-related reactions (fever, chills, itching), heightened susceptibility to infections due to reduced B cell count, and potential long-term impacts on the immune system's ability to combat new infections. As with any medical intervention, patients should consult their healthcare providers to determine the most suitable course of action based on their specific medical situation.

Recently, rituximab has shown promise as a treatment for IgG4-RD. The study conducted in 2019 shows a comprehensive analysis of the safety and effectiveness of rituximab for the treatment of immune-mediated illnesses such as IgG4-RD [24]. A significant number of patients reportedly went into remission after receiving rituximab, and disease activity was significantly decreased. The failure of remission induction by glucocorticoids alone or in conjunction with immunosuppressive drugs was also studied in 2018 [29]. Patients with IgG4-RD who did not improve with standard treatment were shown to have better results when treated with rituximab.

Maintenance treatment with rituximab for IgG4-RD has also been studied for its potential benefits. Wolfson and Hamilos [20] highlighted rituximab as a viable long-term therapeutic option after reviewing recent improvements in understanding and treating IgG4-RD. The advantages of combined treatment with leflunomide and glucocorticoids for remission in IgG4-RD patients were shown in a retrospective analysis and literature review [37].

Rituximab has shown potential in the management of IgG4-RD, although the best therapy method is still up in the air. As Brito-Zerón et al. pointed out, further research is needed to create standardized treatment regimens after they comprehensively examined the therapeutic approach to IgG4-RD [23]. In a network meta-analysis, Omar et al. compared the efficacy of rituximab to that of glucocorticoids and steroid-sparing medicines for IgG4-RD, and they showed that rituximab was superior at producing remission [25].

The findings from the systematic review by Choi et al. and the prospective study by Wang et al. consistently support the efficacy of rituximab in inducing remission and controlling disease activity in IgG4-RDs [27,29]. Other studies, including those by Eroglu et al., Gillispie et al., and Jalilian et al. also reported successful outcomes with rituximab treatment in various manifestations of IgG4-RD [31-33].

Moreover, Wallace et al. identified rituximab as a predictor of disease relapse in IgG4-RDs [28]. This suggests that rituximab provides initial remission and helps prevent future relapses, indicating its long-term therapeutic value. Relapse prediction in IgG4-RD is important for guiding treatment decisions. Elevated blood IgG4 levels were one of the indicators of illness recurrence after rituximab therapy was discovered [28]. Monitoring IgG4 levels may help in forecasting disease activity and directing treatment options, as was observed by Choi et al., who also discovered that blood IgG4 levels during first therapy were predictive of recurrence [27].

Rituximab use has demonstrated potential as both an induction and maintenance therapy for IgG4-RD, particularly in non-responsive cases. It has shown efficacy in inducing remission and improving clinical outcomes. Monitoring blood IgG4 levels during treatment could aid in predicting disease recurrence. However, refining treatment protocols, optimizing rituximab dosing, and identifying predictors of response require additional investigation.

IgG4-RD often impacts multiple organ systems and has a variety of clinical characteristics, which often complicates its diagnosis and treatment. The diagnostic features of IgG4-RD include elevated serum IgG4 levels, eosinophilia, malignant lesions, and proliferation of IgG4-positive plasma cells. This disease can affect any organ, such as the pancreas, lacrimal glands, orbits, retroperitoneum, salivary glands, lymph nodes, lungs, kidneys, and others.

Diagnostic tools including MRI, PET, and CT scans may demonstrate evident signs of fibrosis in the tissue and edema, consequently helping in early diagnosis and to identify the organs that are impacted. However, these diagnostic methods are not definite but they can still aid in early diagnosis of this complex disease. Due to the disease's broad symptomatology and tendency to resemble other autoimmune conditions or malignancies, physicians have to maintain a high index of suspicion.

**Figure 2:**
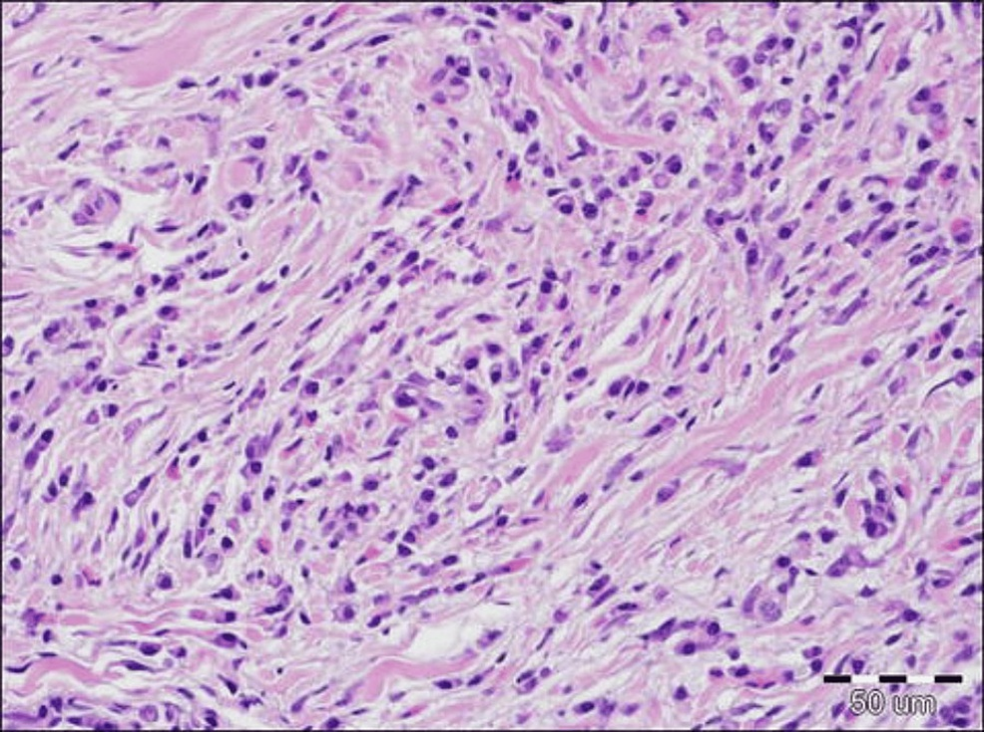
Figure shows extensive fibrosis with storiform appearance and chronic inflammatory infiltrate (hematoxylin & eosin, 200×)

IgG4-RD is recognized by characteristic histopathological markers, including lymphoplasmacytic infiltration, storiform fibrosis, obliterative phlebitis, and an abundance of IgG4-positive plasma cells. These features aid in distinguishing the disease from others and play a pivotal role in its diagnosis. Figure 2 depicts histopathology demonstrating storiform fibrosis [38].

The clinical manifestations of IgG4-RD have been examined in several research. A study in 2017 outlined the diagnostic and therapeutic process for IgG4-RD, stressing the significance of spotting the disease's multi-organ involvement and signature histological features [15]. Al-Mujaini et al. emphasized the wide range of patient presentations and the necessity for a thorough diagnostic strategy [17]. Another study in 2020 found that there was a significant association between the use of rituximab and a high response rate and decreased glucocorticoid dependence in individuals who were unresponsive to first-line treatments [25].

The disease also presents as organ-dependent clinical symptoms, like in autoimmune pancreatitis, patients may experience abdominal pain, jaundice, and weight loss. There can be mucus gland involvement, which can present as xerostomia and sicca syndrome. Chest discomfort, dyspnea, and cough could be symptoms of respiratory tract involvement. A multidisciplinary approach is needed for accurate diagnosis because each afflicted organ system may present with different symptoms.

Clinical similarity of IgG4-RD with other conditions, such as malignancies, granulomatous diseases, and even Sjögren's syndrome, might result in an incorrect diagnosis. The uncommon nature of this condition and the lack of clear clinical indicators frequently result in a delayed diagnosis. When patients report recurring symptoms or unclear multi-organ involvement, clinicians have to assess IgG4-RD as an alternative diagnosis.

IgG4-RD is known for its relapsing-remitting nature. Even with treatment, patients can experience disease flares that necessitate close monitoring and prompt adjustments in management. The clinical features of relapses may mimic the initial presentation or manifest as exacerbations of pre-existing symptoms, emphasizing the need for rigorous follow-up care.

Relapse prediction in IgG4-RD is important for guiding treatment decisions. Elevated blood IgG4 levels were one of the indicators of illness recurrence after rituximab therapy was discovered [28]. Monitoring IgG4 levels may help in forecasting disease activity and directing treatment options was observed by Choi et al., who also discovered that blood IgG4 levels during first therapy were predictive of recurrence [27].

It is important to note that the clinical features can vary depending on the organs affected by IgG4-RD. Additionally, individual cases may present with unique manifestations. Therefore, a comprehensive evaluation of clinical and laboratory findings is necessary for accurate diagnosis of IgG4-RD.

Reporting

The findings of this systematic review are presented in compliance with PRISMA guidelines. A clear and comprehensive representation of the findings is used, and suitable tables or figures are provided to illustrate the essential findings and conclusions from the studies that have been included.

Ethical considerations

As this review is solely based on previously published studies, there is no need for ethical approval for this study. Additionally, there will be no gathering or examination of patient-specific data, ensuring their privacy and confidentiality. The study's focus solely on published literature ensures adherence to ethical standards without compromising data security.

## Conclusions

In conclusion, based on the extensive analysis of literature and studies presented in this review, we affirm that rituximab holds considerable promise as a remarkably effective treatment for IgG4-RD, particularly in cases of relapse and maintaining remission. Despite variable responses observed in some cases, rituximab's overall impact on reducing IgG4 levels, mitigating disease relapse, and sparing the need for high-dose steroids presents a favorable patient profile. Studies reveal that rituximab has shown a superior response when used in combination with other medications to treat this disease. IgG4-RD often targets multiple organs, such as the lymph nodes, pancreas, and salivary glands. Rituximab has emerged to be an effective treatment option with a relatively good safety profile for treating the clinical symptoms associated with this autoimmune disease. This review suggests that, although glucocorticoids remain the first line of treatment for IgG4-RD, rituximab has the potential to become one of the major treatment options for IgG4-RD. Further clinical trials and studies are warranted to maximize its use and determine the long-term benefits of rituximab in IgG4-RD treatment.
